# Polyimide mesh-based sample holder with irregular crystal mounting holes for fixed-target serial crystallography

**DOI:** 10.1038/s41598-021-92687-x

**Published:** 2021-06-23

**Authors:** Ki Hyun Nam, Jihan Kim, Yunje Cho

**Affiliations:** Department of Life Sciences, Pohang University of Science and Technology, Pohang, Gyeongbuk 37673 Republic of Korea

**Keywords:** X-ray crystallography, Nanocrystallography

## Abstract

The serial crystallography (SX) technique enables the determination of the room-temperature structure of a macromolecule while causing minimal radiation damage, as well as the visualization of the molecular dynamics by time-resolved studies. The fixed-target (FT) scanning approach is one method for SX sample delivery that minimizes sample consumption and minimizes physical damage to crystals during data collection. Settling of the crystals on the sample holder in random orientation is important for complete three dimensional data collection. To increase the random orientation of crystals on the sample holder, we developed a polyimide mesh-based sample holder with irregular crystal mounting holes for FT-SX. The polyimide mesh was fabricated using a picosecond laser. Each hole in the polyimide mesh has irregularly shaped holes because of laser thermal damage, which may cause more crystals to settle at random orientations compared to regular shaped sample holders. A crystal sample was spread onto a polyimide-mesh, and a polyimide film was added to both sides to prevent dehydration. Using this sample holder, FT-SX was performed at synchrotron and determined the room-temperature lysozyme structure at 1.65 Å. The polyimide mesh with irregularly shaped holes will allow for expanded applications in sample delivery for FT-SX experiments.

## Introduction

Serial crystallography (SX) using an X-ray free electron laser or synchrotron X-ray is an emerging technique for determining room-temperature structures with minimal radiation damage^1–3^. These room-temperature structures exhibit more biologically relevant structural flexibility compared to traditional cryocrystallography, and can provide reliable structural information on radiation-sensitive proteins^4–6^. Moreover, the SX technique enables visualization of time-resolved structural dynamics in pump-probe studies using optical laser or diffusion-based approaches^7–9^. Therefore, SX can be used to improve the current understanding of the biological and chemical mechanisms of macromolecules compared to conventional X-ray crystallography.

In general SX experiments, X-ray exposes to each crystal sample only once or to renew volumes each time on a large crystal sample^10^. Therefore, large number of crystals are required for collecting complete diffraction data, which is one of the obstacles in SX experiments. Accordingly, to reduce the efforts in sample preparation, minimizing the consumption of crystal samples during SX data collection is one of the key factors. Various sample delivery techniques such as injectors^11–13^, syringes^14,15^, injection with viscous medium^12,14,16–21^, fixed target scanning^22–28^, microfluidic devices^29^, capillaries^5,30^, and conveyer belts^31^ have been developed and applied to SX experiments at XFEL facilities or synchrotrons. Among these, the fixed target sample scanning method has a great advantage for low sample consumption and for minimizing the physical damage to the crystal during the data collection when compared with other sample delivery methods^22,25,26^. Various types of sample holders such as silicon nitride^22,32^, graphene-based chips^33^, and nylon mesh^26,28^ have been developed to stably fix crystals to the sample holder. These crystal supports are regularly arranged with holes of a specific shape and size. During data collection, X-rays are passed through the hole in which the crystal in the sample holder is placed. Transmission of X-rays to crystals through regular holes is advantageous for lowering the background scattering but requires precise alignment of the X-rays and holes. In contrast, in a nylon mesh-based sample holder composed of nylon and polyimide, which can transmit X-rays and generate low background scattering, data is collected by scanning the nylon mesh holes as well as the nylon mesh material on which the crystals can be placed^26,28^. Methods for fixing crystals between Mylar films^34^ and for placing crystals in polyimide tubing^35^ have been applied in FT-SX data collection. Although these methods have been successfully applied in SX experiments, crystals spread onto regular-shaped holes on the sample support or settle in an identical plane in the sample holder, and thus settle in a preferred orientation depending on the shape of the crystals and crystal supporting materials used. If most of the crystals are in the preferred orientation in the sample holder, it may not be possible to collect the complete diffraction dataset or it may be necessary to collect more images compared to a normal dataset.

To obtain crystals in various orientations within the sample holder, a method for supporting crystals embedded in a viscous material between two films was applied for FT-SFX^27^. In this method, lysozyme and glucose isomerase crystals were embedded in agarose and gelatin, respectively, and placed at a thickness of 350 µm between polyimide films^27^. Although this method was effective, it may be difficult to apply when the crystals are physically damaged during the mixing step with viscous medium^27^. On the contrary, FT-SSX with oscillation has been developed to collect the complete dataset by introducing oscillations in the sample holder for a short time when the fixed sample is exposed to X-rays^36^. The advantage of this method is that it can yield diffraction data for a wide area of reciprocal space of the crystal during data collection^36^. However, additional devices are required to apply this system to an existing beamline hutch and the previous beamline equipment needs to be reinstalled.

To enhance the random orientation of crystals on the sample holder, we developed a polyimide mesh-based crystal support with irregular holes for SX experiments. The irregular hole in the polyimide film was fabricated using a picosecond laser. Using this crystal support, we performed fixed-target serial synchrotron crystallography (FT-SSX) experiments using lysozyme crystals of 30–40 µm and determined its room-temperature structure at 1.65 Å. This polyimide mesh-based crystal support with irregular holes can be used with the sample holder in FT-SX experiments.

## Results

### Fabrication of polyimide mesh with irregular holes

We used the following criteria to develop a new crystal support for FT-SX: (1) The crystal support material should transmit X-rays. (2) X-ray background scattering from the crystal support should not affect data analysis. (3) During data collection, precise alignment between the X-ray and sample holder should not be required. (4) To increase random orientations of the crystal, the hole in which the crystal is mounted and its surrounding environment, should be irregular. (5) The sample holder should maintain the hydrated environment of the crystal sample.

**Figure 1:**
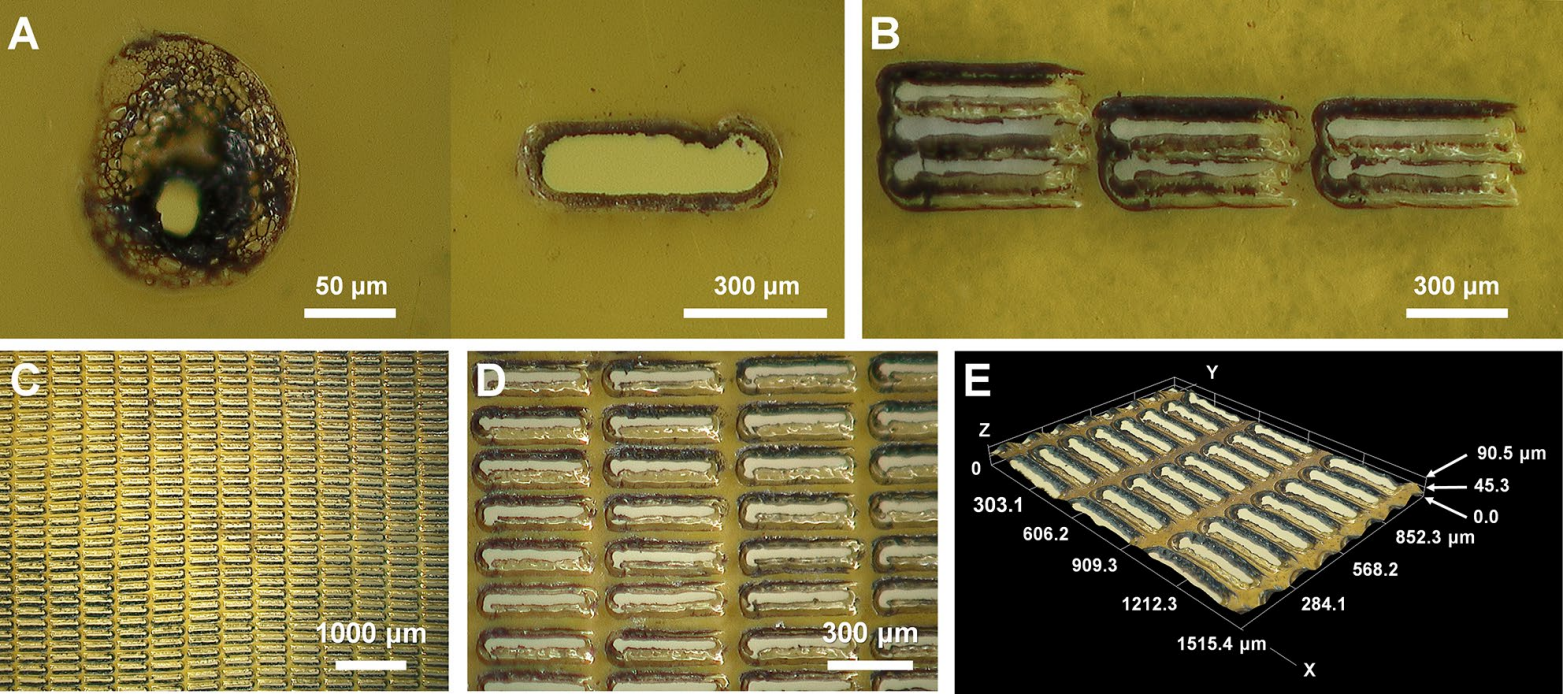
Fabrication of a polyimide-mesh using a nanosecond laser. (**A**) Thermal damage on the polyimide film by laser drilling. (**B**,**C**) Fabricated polyimide mesh with irregular holes created using a laser. (**D**,**E**) Close-up view of the fabricated polyimide mesh.

Polyimide film was selected as the crystal-supporting material because it satisfied our sample holder development criteria. Initially, we considered fabricating holes with various shapes in a single polyimide film by using a laser drilling method to provide a random orientation when the crystals are mounted on the holes in the polyimide film. During the initial laser drilling experiment on the polyimide film, we observed that thermal damage occurred around the location where the laser penetrated (Fig. 1A). Even if lasers with the same spacing and power were used to penetrate the polyimide layer, drilling resulted in irregular shaped holes because of the inherent thermal damage at the laser drilling point. We considered that this feature was very useful for enabling the random orientation of crystals on the polyimide mesh. Next, laser drilling in a rectangular shape was performed on the polyimide film. During laser drilling of the polyimide film, when the drilling interval was narrow, the film spacer between the drilling holes was thermally damaged. To prevent this, a polyimide mesh with an irregular hole shape was manufactured by controlling the laser energy intensity and laser transmission position (Fig. 1B). Finally, the intensity and exposure position of the laser were adjusted so that the hole size was maintained at approximately 350 × 50 μm (Fig. 1C). As observed under a microscope, the holes were rectangular with rounded corners (Fig. 1D). The shape of each of the pores in the polyimide mesh was varied due to thermal damage, which would allow the crystals to be settled in a relatively random manner compared to a sample holder with holes of uniform shape. The thermally damaged film showed an irregular depth of approximately 20–30 μm in the rear direction through which the laser penetrated (Fig. 1E). These crystal supporting holes formed an irregularly shaped funnel-like structure. When spreading the crystals on the polyimide mesh-based sample support with irregular holes, the crystals could be settled on the polyimide mesh surface and inside the hole. The distance of the crystal position from the surface of the polyimide mesh to the inside of the hole was less than 50 μm, which was in a range that does not critically affect the distance from the sample to the detector during data processing.

### Assembly of the sample holder

**Figure 2:**
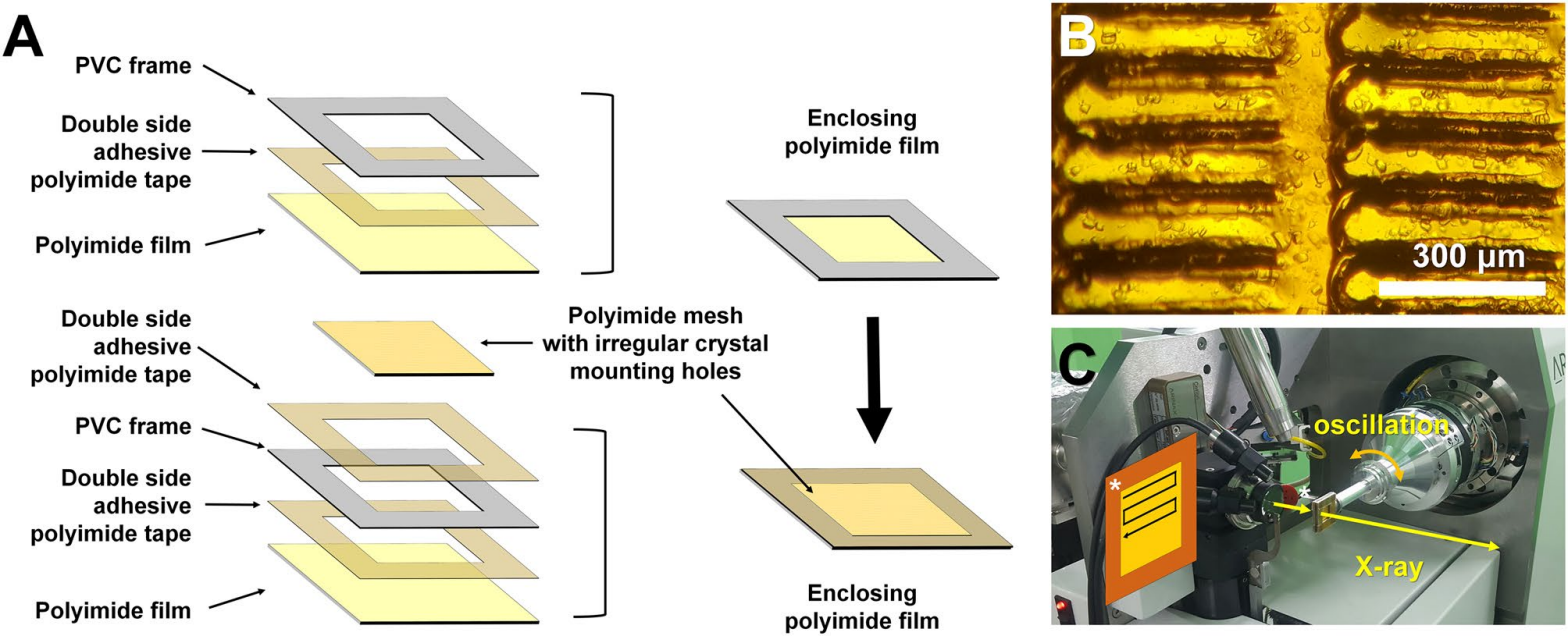
Preparation and application of the polyimide mesh-based sample holder with irregular crystal mounting holes. (**A**) Assembly of the polyimide mesh with irregular crystal mounting holes containing crystals enclosed with film to prevent dehydration. (**B**) Microscopic view of lysozyme crystals mounted on the polyimide mesh. (**C**) Experimental setup of the polyimide mesh-based sample holder with irregular crystal mounting holes on the goniometer for FT-SX.

When crystals are spread on a polyimide mesh and then exposed to room temperature in the absence of hydration, a dehydrated environment is created by evaporation of the crystallization solution. This may cause various problems, such as changes in the crystal space group, collapse of the crystal lattice, and deterioration of data quality because of salt crystallization from the evaporated crystal solution^26,37^. To prevent these effects, it is very important to maintain the hydrated environment of the crystals settled on the polyimide mesh. In this experiment, the method of enclosing the mounted crystal sample using a polyimide film was the same as that for the previously used nylon mesh-based sample holder^26,28^. The enclosing polyimide film was attached to the PVC frame for easy handling (Fig. 2A and Supplementary Figure S1). When a crystal-mounted polyimide mesh was placed between the polyimide films, the bottom and upper films were enclosed using double-sided adhesive polyimide film (Fig. 2A,B and Supplementary Figure S2). This sample holder was attached to a magnetic base and then mounted on the goniometer for data collection by the raster scanning method (Fig. 2C).

### Application of the polyimide-mesh based sample holder to FT-SSX

To demonstrate the application of the polyimide mesh-based sample holder with irregular crystal mounting holes, FT-SSX experiments were performed using lysozyme as a model sample on an X-ray synchrotron. Data were collected by raster scanning with oscillation at room temperature (Table 1). Although the X-ray size at the sample position was less than 10 μm (full-width half maximum) in both the horizontal and vertical directions, to avoid radiation damage to neighboring crystals around the X-ray penetration point, the interval of raster scanning points was selected as 50 μm in both the vertical and horizontal directions^38^. During raster scanning, the sample holder was moved both vertically and horizontally by 6750 μm. Accordingly, there were 135 scan points in the both vertical and horizontal directions, and 36,450 images were collected in 80 min using two sample holders. At each raster scanning point, the X-ray exposure time was 100 ms with an oscillation of 0.011°. The average diffracted weight dose and the average dose to the exposed area of the crystal for the X-ray used in this experiment were calculated as 0.607264 MGy and 0.309161 MGy, respectively. Further, the recent experimentally determined dose limit was 0.38 MGy when performing the room-temperature SSX experiment^38^.

**Table 1:** Data collection and refinement statistics.

Data collection	Lysozyme
Energy (eV)	12,659
Expose time (ms)	100
Space group	P4_3_2_1_2
**Cell dimension (Å)**	
a	79.45
b	79.45
c	38.47
Collected images	36,450
Hits images	11,958
Indexed images	7288
Indexed pattern	7423
Resolution (Å)	80.0–1.65 (1.71–1.65)
Unique reflections	15,398 (1498)
Completeness (%)	100.0 (99.93)
Multiplicity	83.7 (57.7)
SNR	6.58 (1.58)
CC	0.9836 (0.5635)
CC*	0.9958 (0.8490)
R_split_ (%)^a^	10.69 (70.26)
Wilson B factor (Å^2^)	31.76
**Refinement**	
Resolution (Å)	56.18–1.65 (1.70–1.65)
R_work_^b^	17.85 (27.74)
R_free_^c^	20.87 (27.92)
R.m.s. deviations	
Bond length (Å)	0.013
Bond angle (°)	1.618
B factors (Å)	
Protein	31.23
Water	34.03
Ramachandran (%)	
Preferred	98.43
Allowed	1.57
Outliers	0.00

Highest resolution shell is shown in parentheses.

^a^*R*_*split*_ = $$\left( {{\raise0.7ex\hbox{$1$} \!\mathord{\left/ {\vphantom {1 {\sqrt 2 }}}\right.\kern-\nulldelimiterspace} \!\lower0.7ex\hbox{${\sqrt 2 }$}}} \right)\cdot\frac{\sum _{hkl}\left|{I}_{hkl}^{even}-{I}_{hkl}^{odd}\right|}{\frac{1}{2}\left|{I}_{hkl}^{even}-{I}_{hkl}^{odd}\right|}$$.

^b^*R*_work_ = Σ||*F*_obs_| − |*F*_calc_||/Σ|*F*_obs_|, where *F*_obs_ and *F*_calc_ are the observed and calculated structure-factor amplitudes respectively.

^c^R_free_ was calculated as R_work_ using a randomly selected subset of unique reflections not used for structure refinement.

**Figure 3:**
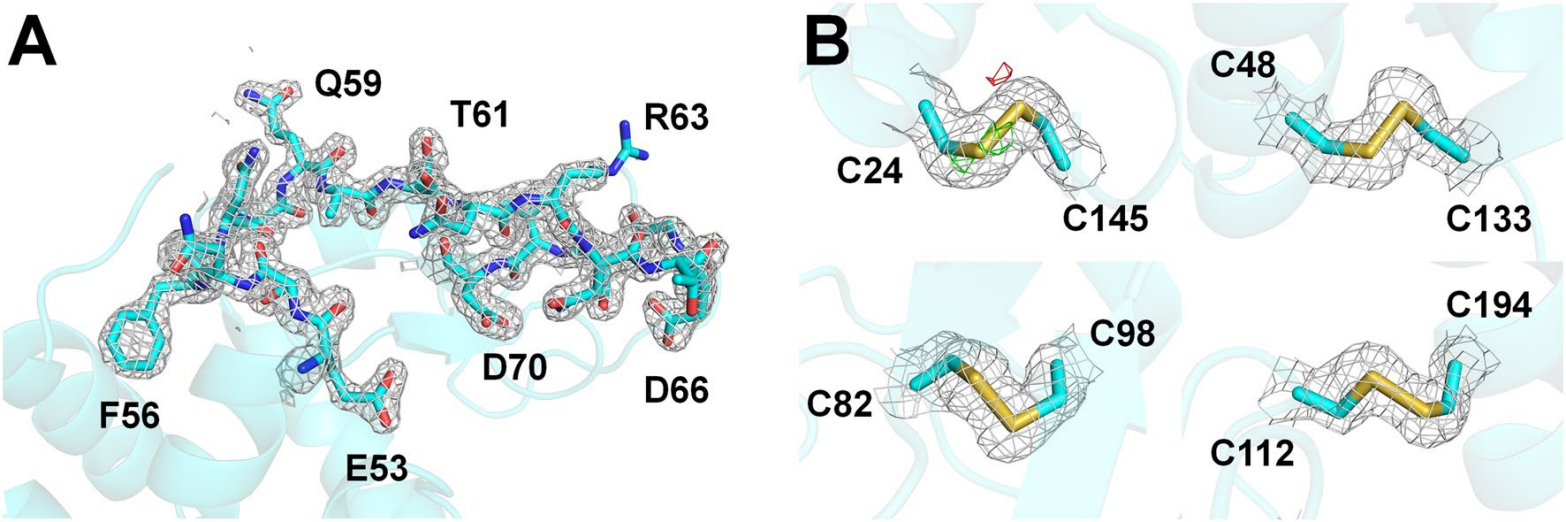
Electron density map of room-temperature lysozyme supported by the polyimide mesh-based sample holder. (**A**) 2mFo-DFc electron density map (gray mesh, 1.2 σ) of the active sites (Asp56 and Asp72) of lysozyme. (**B**) 2mFo-DFc (gray mesh, 1.2σ) and mFo-DFc (green mesh, + 3 σ; red mesh, − 3 σ) electron density map for the disulfide bonds in lysozyme.

In total, 11,958 images included Bragg peaks with a hit rate of 32.80%. Through indexing, 7423 lysozyme diffraction patterns were obtained from all 7288 hit images. The indexing and multi-crystal hit rates were 60.94% and 1.85%, respectively. Data were collected up to 1.65 Å with a completeness of 100%, and the redundancy, CC, CC*, SNR, and R_split_ were 83.7, 0.9836, 0.9958, 6.58, and 10.69, respectively. Structure refinement was performed to 1.65 Å, and the R_work_ and R_free_ of the final model were 17.85% and 20.87%, respectively. A clear electron density map was observed from Lys19 to Leu145 (Fig. 3A). Electron density map analysis showed no significant negative Fo-Fc electron density, considered as radiation damage, at the disulfide bond sites (Csy24-Csy145, Csy48-Csy133, Cys82-Cys98, and Cys94-Cys112) (Fig. 3B), which are relatively more radiation-sensitive than other amino acids. Superimposition of the lysozyme delivered in the polyimide mesh with the previously reported lysozyme structure at room temperature using a nylon mesh (PDB code 6IRJ), polyacrylamide (6I6G), shortening (6KCB), wheat starch (7BVM), and lard injection matrix (7CJZ) revealed a high similarity with a root mean squared deviation of 0.106–0.231 Å for all Cα atoms.

**Figure 4:**
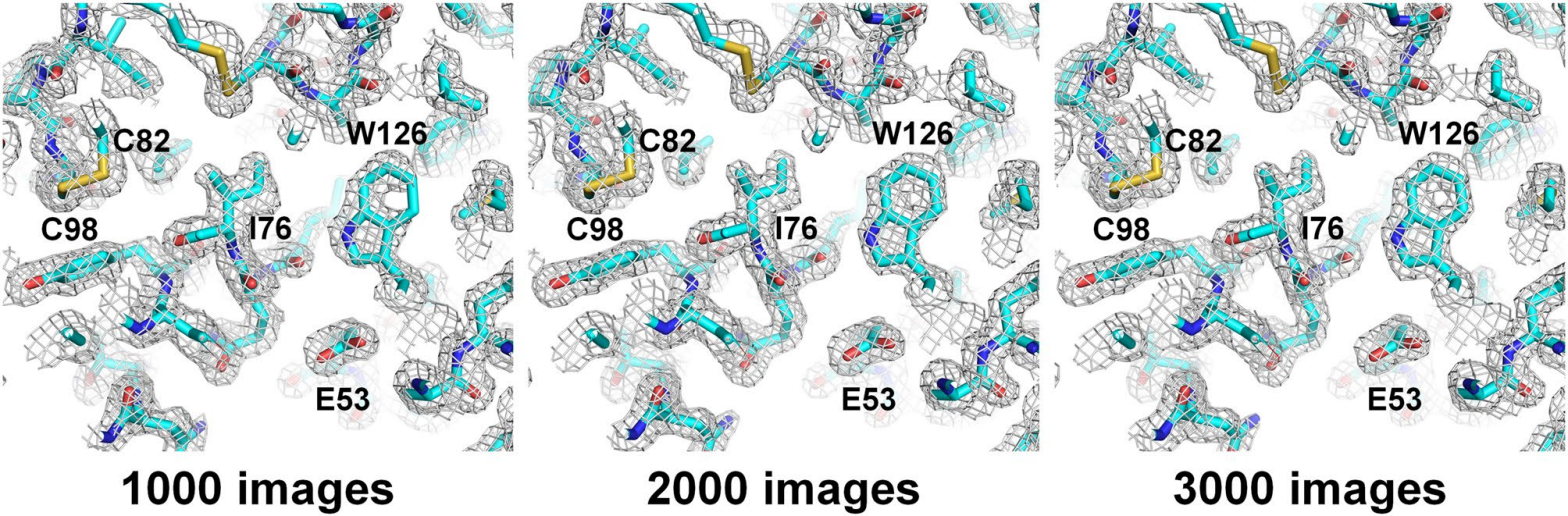
2mFo-DFc electron density map (gray mesh, 1.3 σ) for lysozyme datasets merged from (**A**) 1000, (**B**) 2000, and (**C**) 3000 images.

Next, we re-processed the collected data to analyze whether minimal images were required to obtain a reliable structure. Following processing with 1.65 Å resolution in 1000 units (Table 2), when 3000 diffraction patterns were used, the overall completeness, SNR, CC, CC*, and R_split_ were 99.96, 4.25, 0.9277, 0.9810, and 21.50, respectively. Although the quality of the data statistics was low compared to that of the complete data, we obtained a dataset suitable for structure determination from at least 3000 diffraction patterns. In further data processing, datasets merged from 1000, 2000, and 3000 images provide the solution for molecular replacement. Although all data showed a reliable electron density map (Fig. 4), only the dataset merged from 3000 images provided the appropriate model with a R_work_/R_free_ of 0.2162/0.2599.

**Table 2:** Data collection and refinement statistics.

Data processing	No. of indexed images		
	1000	2000	3000
Resolution (Å)	80.0–1.65 (1.71–1.65)		
Unique reflections	14,827 (1413)	15,355 (1490)	15,398 (1499)
Completeness (%)	96.29 (94.26)	99.72 (99.40)	99.96 (99.80)
Multiplicity	8.7 (5.9)	17.8 (12.1)	28.9 (19.7)
SNR	3.00 (1.13)	3.43 (1.28)	4.25 (1.61)
CC	0.8116 (0.3654)	0.8792 (0.4508)	0.9277 (0.5570)
CC*	0.9465 (0.7316)	0.9673 (0.7883)	0.9810 (0.8458)
R_split_ (%)	37.53 (110.47)	28.69 (81.67)	21.50 (67.10)
**Molecular replacement**			
Top LLG	3361.874	5053.713	6122.978
Top TFZ	53.6	60.1	62.4
**Refinement**			
Resolution (Å)	56.18–1.65 (1.73–1.665)		
R_work_	0.2993 (0.4063)	0.2500 (0.3357)	0.2162 (0.3104)
R_free_	0.3563 (0.4110)	0.2935 (0.3977)	0.2599 (0.3463)

### Measurement of background scattering

**Figure 5:**
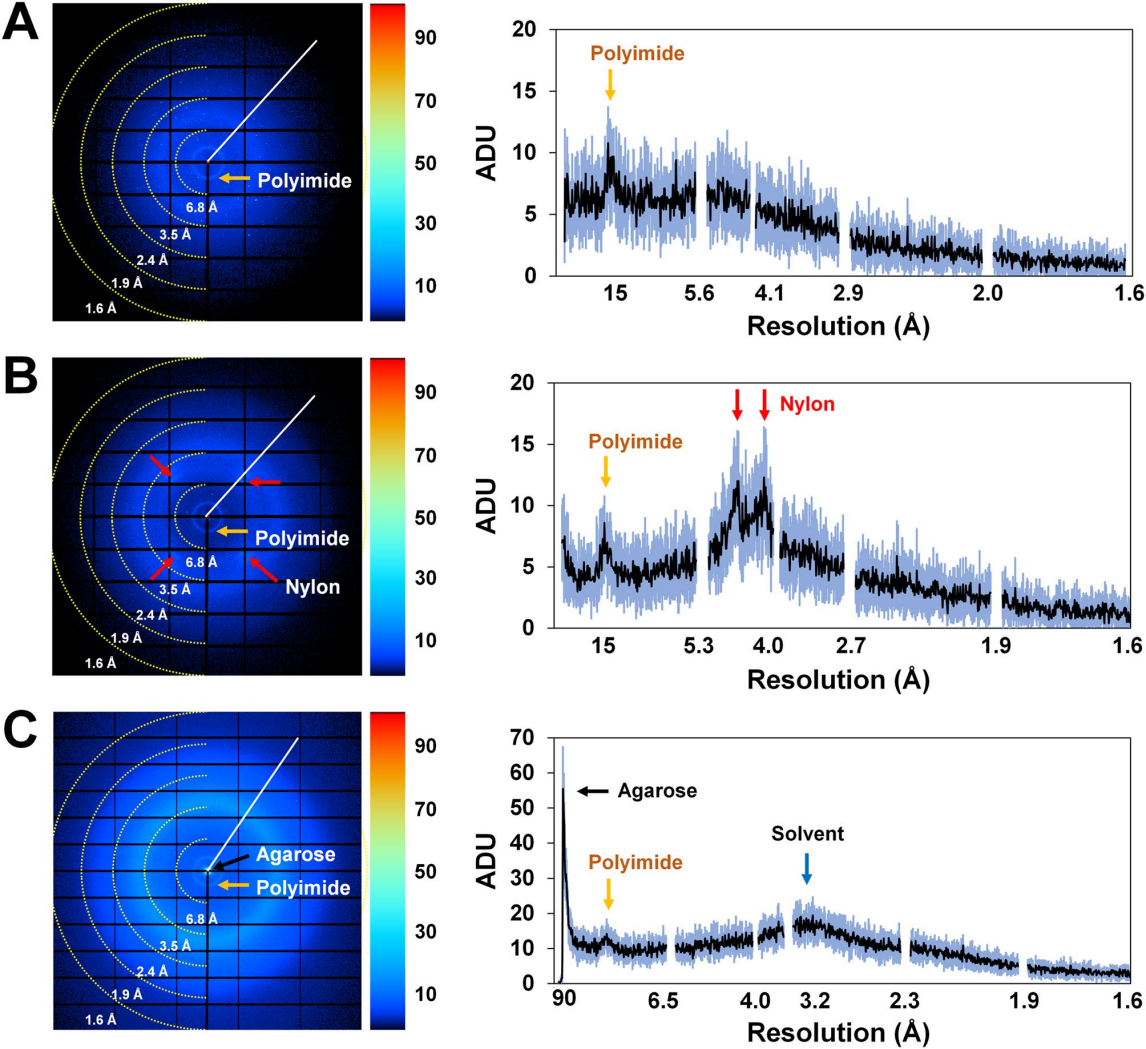
Background scattering analysis. (**A**) Polyimide mesh-based sample holder. (**B**) Nylon mesh-based sample holder. (**C**) Viscous (agarose)-medium-based crystal support in a sample holder. The 2D profiles of average and standard deviations of X-ray background scattering are indicated by black and blue, respectively.

Polyimide is well-known to generate very low background scatter by X-rays; in this experiment, polyimide showed negligible levels of X-ray scattering during data processing^39^. Nevertheless, analysis of background scattering from polyimide generated during data collection is essential for further SX applications. In this experiment, the thickness of the polyimide mesh and polyimide film used for enclosing the film was 25 μm. As the data were collected by raster scanning, X-rays penetrated the polyimide mesh pores, as well as the polyimide mesh, and the thicknesses through which X-rays were transmitted were 50 and 75 µm, respectively. Because both materials were composed of the same polyimide, they both exhibited circular background scattering at 15 Å (Fig. 5A). The thickness of the 50- and 75-μm polyimides showed 7–9 analogue-to-digital units (ADUs) (Fig. 5A). We consider this value to indicate a very low background scattering that is negligible in data processing. Next, we compared background scattering of the polyimide mesh-based sample holder with the previously reported nylon mesh and viscous-based background scattering. The background scattering of nylon mesh showed 10 ADU at 3.4 and 4.5 Å, respectively, which included 7 ADU at 15 Å generated from the enclosing polyimide film (Fig. 5B). For the crystal support using agarose, background scattering from agarose showed 55 ADU at 90 Å, and diffuse background scattering for the solution contained ~ 15 ADU at 3.2 Å (Fig. 5C). This support was also enclosed by a polyimide film and showed ~ 13 ADU at 15 Å (Fig. 5C). Although background scattering from the nylon mesh and from the viscous materials did not significantly affect data processing, the polyimide mesh-based sample holder showed the least background scattering.

## Discussion

We developed a new crystal support using a polyimide mesh-based sample holder with irregular crystal mounting holes for application in FT-SX. Using this sample holder, we successfully determined the room-temperature structure of lysozyme FT-SSX. General sample holders have the same size and shape of holes in which the crystal is mounted; we fabricated a sample holder with holes showing irregular shapes based on thermal damage that occurs when the laser penetrates the polyimide film. It was not possible to directly compare and analyze the extent to which the polyimide mesh with irregular holes conferred significant advantages in data collection. However, when a crystal is placed in a regular-shaped hole and the sample holder is placed vertically for data collection, it will be placed in the preferred orientation according to the shape of the crystal and the hole of the sample holder when the crystal is settled at the bottom of the hole by gravity. In contrast, for the irregular hole applied in this experiment, even if the crystal sinks to the bottom of the hole via gravity, it settles in a random orientation because the bottom of the holes is irregular.

In previously reported FT-SX, sample mounting methods such as a sandwich and sheet-on-sheet approach do not require precise alignment between the sample holder and X-ray^23,34^; therefore, they do not require technical operation and allow more data collection during beamtime. As all the material in the sample holder used in this study is composed of polyimide, through which X-rays can penetrate, it is not necessary to precisely align the X-rays and sample holders, in contrast to a sample holder in which the X-rays pass through a specific hole. In addition, the X-ray background scattering generated from the sample holder occurs in the low-resolution region, and thus does not significantly affect data resolution; further, the intensity of X-ray background scattering is extremely low. Accordingly, this sample holder may be applicable for crystal samples with a weak diffraction limit as well as in phasing experiments, where low background scattering is an important factor. In this study, we created irregular holes in the polyimide film using a picosecond laser; however, crystal mounting holes of various shapes can also be made by precision microfabrication or other laser sources. The rectangular crystal mounting hole had a size of 50 × 350 μm, which is suitable for a crystal of 20–50 μm in size. In future application studies, when fabricating a polyimide mesh with irregular holes, the size of the mesh pores should be adjusted considering the purpose of the study and the size of the crystal. Additionally, because the sample holder is composed only of polyimide film, it can be reused without causing physical damage during synchrotron X-ray experiments; in contrast, when applied with an X-ray free electron laser, the polyimide mesh is damaged.

In conclusion, the proposed polyimide mesh-based sample support with irregular crystal mounting holes can be used for future FT-SX studies. However, since this experiment did not quantitatively investigate the effectiveness of the sample holder with irregular holes is for data collection, future studies need to compare the diffraction data quality to that of a traditional sample holder with a flat surface. Specifically, it is necessary to compare the random orientation of the crystals in the two sample holders using a low symmetry crystal, and to compare the number of indexed images required to determine the structure with a certain level of quality (completeness balanced with redundancy). This further study would provide insight into the data collection efficiency of a sample holder with irregular holes by performing a repeated data collection on a number of mounts.

## Methods

### Fabrication of polyimide mesh

The polyimide film (25 μL) was purchased from Covalue Youngjin Co. (Daegu, Republic of Korea). Laser drilling of polyimide to create crystal mounting holes was performed using a picosecond laser at UV 355 nm (Kortherm Science Co., Ltd., Incheon, Korea). The marking speed and delay were 300 mm/s and 310 μs, respectively. The jump speed and delay were 583.3 mm/s and 500 μs, respectively. To remove the dust generated during laser drilling, the polyimide-mesh was rinsed with water and ethanol.

### Protein crystal preparation

Lysozyme from chicken egg white was purchased from Sigma-Aldrich (L6876; St. Louis, MO, USA). Lysozyme was crystallized by the microtube batch crystallization method as reported previously^18^. Briefly, lysozyme powder was dissolved in a buffer containing 10 mM Tris–HCl, pH 8.0, and 200 mM NaCl, and the final lysozyme solution concentration was 50 mg/mL. The protein solution (200 μL) was mixed in a microtube containing crystallization solution (200 µL) composed of 0.1 M Na-acetate, 2 M NaCl, and 8% (w/v) PEG8000. The mixed samples were immediately vortexed for 30 s and then incubated overnight at 20 °C. The crystal size of the lysozyme was 30–40 μm.

### Preparation of sample holder

To facilitate the handling of the enclosing film, a 0.3-mm-thick PVC frame was attached to a polyimide film (13 × 13 mm) with double-sided adhesive polyimide tape. A polyimide-mesh with irregular holes (8 × 8 mm) was placed on the first enclosing film. The lysozyme crystal suspension (30 μm) was transferred onto the polyimide mesh prepared with irregular holes using a pipette. After spreading the crystals with a pipette tip, 15 μL of crystallization solution was removed from the corner of the sample holder, and the second enclosing film was immediately covered with double-sided adhesive polyimide tape. The sample holder was then mounted on the goniometer and fixed to the magnetic base using clay.

### Data collection

The FT-SSX experiment using the polyimide mesh with irregular holes was performed at the 11C beamline at the Pohang Light Source (PLS-II, Republic of Korea). The X-ray energy and photon flux were 12.659 keV and 1.3 × 10^12^ photons/s, respectively. The beam size at the sample position was 4.5 (vertical) × 8 (horizontal) μm^2^ (full-width half maximum). Data were collected as previously reported for FT-SSX using a nylon mesh-based sample holder^28^. Briefly, raster scanning was performed at 50-µm intervals in both the horizontal and vertical directions. The X-ray was exposed to each raster scanning point for 100 ms with 0.011° oscillation. The average diffraction weighted dose and average dose in the exposed region of the crystal were calculated using RADDOSE-3D^40^. Data were collected at room temperature (24–25 °C) and diffraction patterns were recorded on a Pilatus 6 M with a 10-Hz readout (Dectris, Baden-Daettwil, Switzerland).

### Data processing and structure determination

Hit images containing diffraction patterns were filtered using Cheetah^41^ and then processed with CrystFEL^42^. The phasing problem was solved by molecular replacement using phase-MR in Phenix^43^, with lysozyme (PDB code 7CJZ)18 used as the search model. Manual model building was performed using Coot^44^. Structure refinement was performed using Phenix.refinement in PHENIX^43^. The geometry of the final model was validated using MolProbity^45^. Structure figures were generated using PyMOL (https://pymol.org/). The coordinates and structure factors have been deposited to Protein Data Bank under the accession code 7DTB. Diffraction images have been deposited to CXIDB under ID 163.

### Background scattering analysis

The polyimide mesh with irregular holes, nylon mesh-based sample holder, and viscous medium-based crystal supporting sample holder were exposed to an X-ray for 100 ms. The polyimide mesh (25 µm), nylon-mesh (pore size: 60 µm), and gelatin viscous medium (thickness: < 350 µm) were enclosed in two polyimide films (total thickness: 50 µm). The X-ray energy and photon flux were 12.659 keV and 1.3 × 10^12^ photons/s, respectively. Among the collected data, 20 images were randomly extracted, and background scattering was analyzed using ADXV (https://www.scripps.edu/tainer/arvai/adxv.html).

## Supplementary Information

Supplementary Information.
