# Supplementary material for: Polyimide mesh-based sample holder with irregular crystal mounting holes for fixed-target serial crystallography
Source: Sci Rep. 2021 Jun 23;11:13115. doi: 10.1038/s41598-021-92687-x (PMC8222285; doi:10.1038/s41598-021-92687-x)
Supplement: Supplementary file 1 — Supplementary Information. [file 41598_2021_92687_MOESM1_ESM.docx]

**Supplementary Information**

**Polyimide mesh-based sample holder with irregular crystal mounting holes for fixed-target serial crystallography**

Ki Hyun Nam*, Jihan Kim, Yunje Cho

Department of Life Sciences, Pohang University of Science and Technology, Pohang, Gyeongbuk 37673, Korea

* Correspondence. structures@postech.ac.kr (K.H.N.)

Contents

Materials and methods

Supplementary Figures S1-S2

Materials and Methods

**Crystallization**
Thaumatin from *Thaumatococcus daniellii* was purchased from Sigma-Aldrich (T7638; St. Louis, MO, USA). The protein powder was dissolved into a distilled deionized water. The thaumatin solution (100 μl; 40 mg/ml) was mixed with the crystallisation solution (100 μl) containing 0.1M HEPES, pH 7.5 and 1M Na/K tartrate in 1.5 ml microcentrifuge tube, and subsequently mixed using a vortex machine at 3,000 rpm for 10 s. This mixture solution was incubated at 18 °C, and microcrystals appeared after four hours.


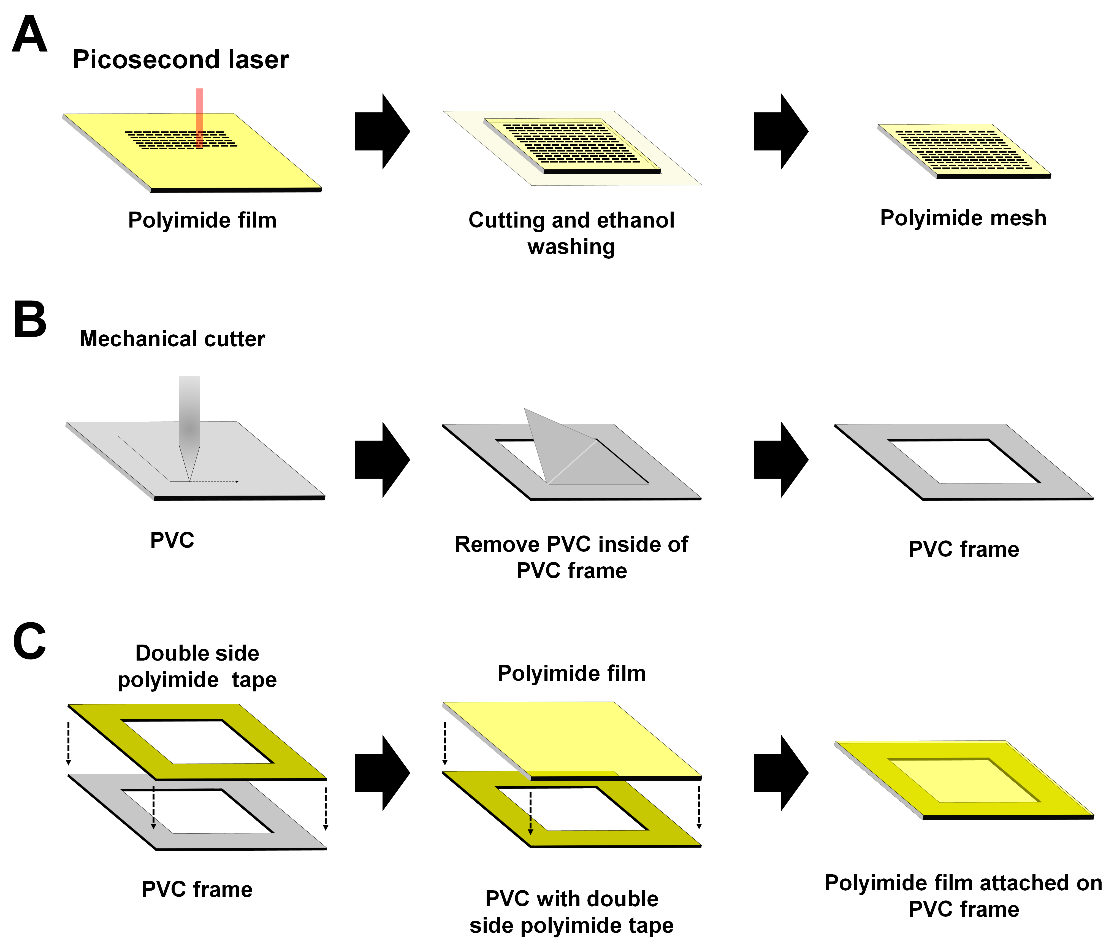


**Supplementary Figure S1**. Fabrication of the polyimide mesh-based sample holder components. (A) Fabrication of the polyimide mesh. After drilling the polyimide film with a picosecond laser, it was washed with ethanol and cut to fit the holder. (B) PVC frame fabrication. After cutting the PVC film with a mechanical cutter, the inner PVC section was removed. (C) Fabrication of polyimide film for the sample holder. A double-side polyimide tape was attached to the PVC frame and then the polyimide film was covered**.**


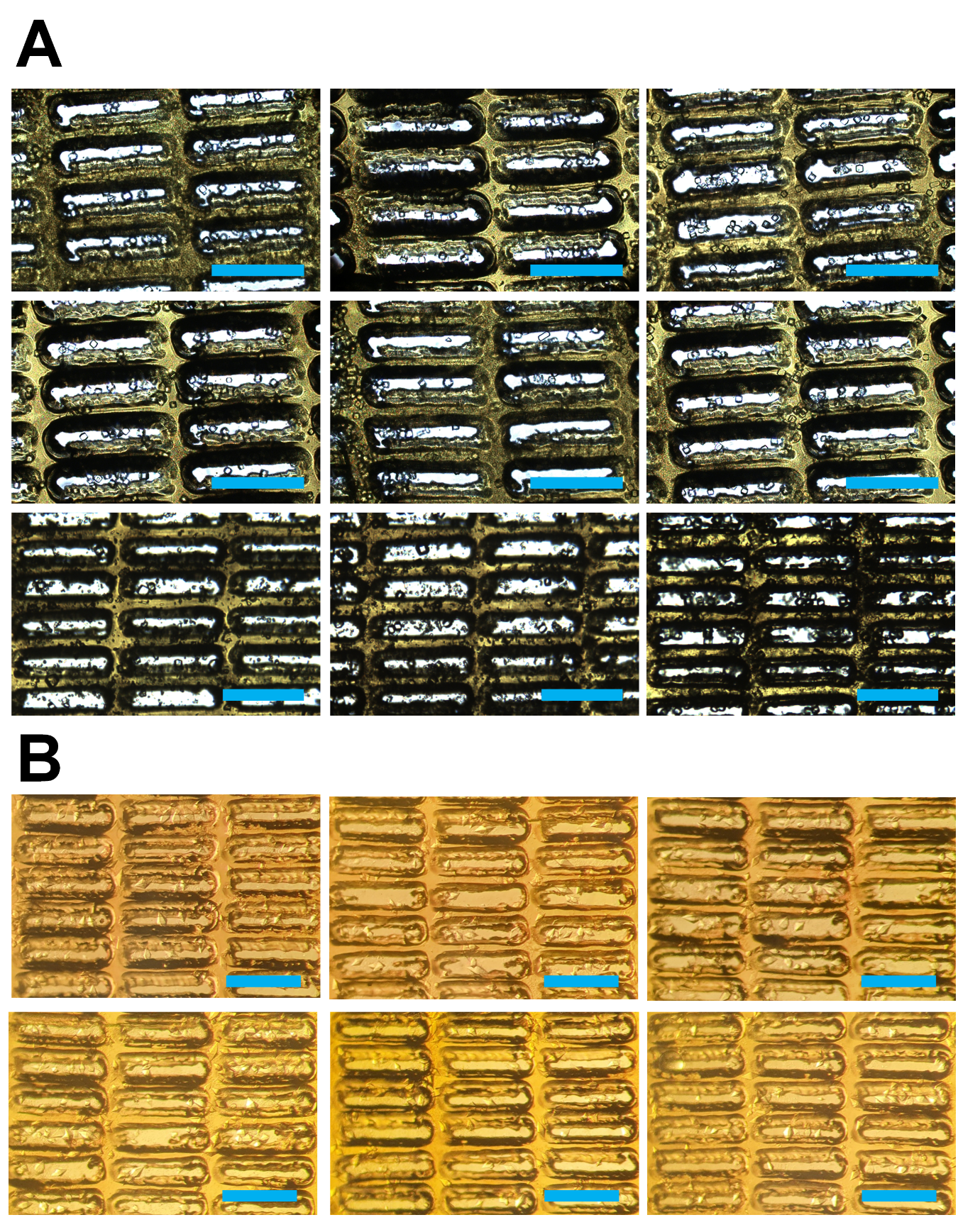


**Supplementary Figure S2**. Images of (A) lysozyme and (B) thaumatin crystals on the polyimide mesh-based sample holder (scale bars: 300 μm).
